# Selective Processing of Multiple Features in the Human Brain: Effects of Feature Type and Salience

**DOI:** 10.1371/journal.pone.0016824

**Published:** 2011-02-09

**Authors:** E. Menton McGinnis, Andreas Keil

**Affiliations:** National Institute of Mental Health Center for the Study of Emotion and Attention, University of Florida, Gainesville, Florida, United States of America; University of Regensburg, Germany

## Abstract

Identifying targets in a stream of items at a given constant spatial location relies on selection of aspects such as color, shape, or texture. Such attended (target) features of a stimulus elicit a negative-going event-related brain potential (ERP), termed Selection Negativity (SN), which has been used as an index of selective feature processing. In two experiments, participants viewed a series of Gabor patches in which targets were defined as a specific combination of color, orientation, and shape. Distracters were composed of different combinations of color, orientation, and shape of the target stimulus. This design allows comparisons of items with and without specific target features. Consistent with previous ERP research, SN deflections extended between 160–300 ms. Data from the subsequent P3 component (300–450 ms post-stimulus) were also examined, and were regarded as an index of target processing. In Experiment A, predominant effects of target color on SN and P3 amplitudes were found, along with smaller ERP differences in response to variations of orientation and shape. Manipulating color to be less salient while enhancing the saliency of the orientation of the Gabor patch (Experiment B) led to delayed color selection and enhanced orientation selection. Topographical analyses suggested that the location of SN on the scalp reliably varies with the nature of the to-be-attended feature. No interference of non-target features on the SN was observed. These results suggest that target feature selection operates by means of electrocortical facilitation of feature-specific sensory processes, and that selective electrocortical facilitation is more effective when stimulus saliency is heightened.

## Introduction

When searching the visual environment for a specific target that is not defined by spatial location, humans often encounter complex, multi-feature stimuli that can be identified only by a combination of their visual properties. For instance, finding one's vehicle in a parking lot cannot be accomplished by focusing on the color alone. The attentive selection of object features at the cost of visual information that does not match a specified multi-feature target has been studied in the laboratory and has been referred to as feature-based selective attention [1], [2]. In a typical feature-based attention paradigm, one or more features may define a target stimulus vis-à-vis a non-target, thus requiring active discrimination of features, e.g., color, motion, orientation, or shape. A variety of processes have been suggested to be involved in identifying a target object based on pre-defined features: First, the visual system needs to enhance the sensitivity to the target features at the cost of competing non-target features. Second, such object features (in the present study: color, orientation, and shape) must be integrated into cohesive entities, forming a percept.

Traditionally, feature-based attention research has examined feature-dependent selection of targets in its interaction with spatial attention [3]. For instance, in Treisman's Feature Integration Theory, spatial selection is a prerequisite for object identification [4], [5], [6], while opposing notions suggest that features are processed in parallel across the visual field [7], [8]. In an effort to characterize the neural mechanisms underlying feature-based selective attention in the absence of spatial cues, animal and human models have been developed to describe the brain regions and the neural timing of feature selection. For instance, the feature similarity gain model, proposed by Treue and Martinez-Trujillo (1999), states that neuronal responses are enhanced for all neurons whose sensory selectivity matches the current attentional state. Empirically, Treue and Martinez-Trujillo (1999) demonstrated that macaque monkeys attending to a random dot pattern moving in a given direction enhanced the responses of neurons whose preferred direction matched the attended direction, while the response for neurons preferring the opposite direction was reduced. In line with such a model, neuroimaging studies have pinpointed cortical activation areas associated with attention to a particular feature [9]. Similar evidence has been provided by early human PET (positron emission tomography) studies [8], [9]; for example, attending to color has been localized to the inferior occipital area V4/V8 [10], [11], [12].

The question arises as to the temporal sequence of processes involved in the selection of different visual features. In addition, it is unclear how target and non-target features interact when embedded in complex stimuli appearing with different feature combinations [13]. The processing of such features can be explored using ERPs, which have a time resolution in the millisecond (ms) range, allowing for an accurate calculation of the temporal characteristics of neural activity.

### ERP studies of feature-based attention

The comparison of ERPs in response to target features against ERPs in conditions with no target features typically results in the modulation of a broad posterior negative-going ERP, termed selection negativity (SN; [13]), which often begins at the peak of the visual N1 component, i.e., at around 160 ms post-stimulus. This index is best observed in difference waves obtained by subtracting the ERP responses for items with targets features from those with less or no target features [14], [15]. The resulting SN waveform has been shown to systematically correlate with the discrimination and selective processing of target features. Temporally, the SN typically begins between 140–180 ms post-stimulus and continues for an additional 200 ms [13]. Using subtractions of conditions in which the same stimulus was viewed under different directions, it is possible to identify the residual ERP that corresponds with any given feature-attention combination. Importantly, this can be calculated for non-target stimuli, in which no target-related response is made. When using multi-feature stimuli, the selection of a given feature (e.g., color) can therefore be examined in the context of different numbers (levels) of other task-relevant features. For instance, the ERP difference of target color versus non-target color can be calculated for items that have all, some, or no other target feature.

Both the latency and amplitude of the SN are sensitive to the nature of the target features [1], and to the number and discriminability of features [16]. In line with such a notion, the addition of target features to a compound stimulus may delay the selection process, thereby increasing the SN time window [17]. Taken together, this previous work converges with animal models of feature-based attention suggesting that selection affects perceptual processes and that the selection process varies as a function of the ongoing sensory process.

The P3 component of the ERP is probably the most frequently examined event-related electrophysiological variable and has been suggested to index a variety of cognitive and behavioral processes [18] [19]. Relevant for the present research, the P3 is sensitive to processes linking higher-order perceptual analysis to response initiation [20], for instance when participants classify task-relevant stimuli. The P3 is denoted by a positive voltage deflection typically occurring between 300–600 ms post-stimulus and is a valid indicator of the extent to which a given stimulus bears similarity to the target. It is also well known that attention to non-spatial features affects the ERP waveform at a later point in time than spatial features, and later than the SN time range [21], [22], [23], [24], [25], [26], [27], [28]. The modulation of the P3 by different feature combinations thus allows us to establish to what extent specific feature combinations impact the classification of a stimulus as the target versus non-target. For example, in a feature discrimination task using color and form as cues, larger positivity was demonstrated in the P3 time window for target stimuli, but only when attention was directed to stimulus color [29]. These data suggest that the mechanisms underlying selective attention to color differ from the mechanisms responsible for the attentional selection of other non-spatial attributes, such as stimulus form. Additionally, the P3 can serve as an index of task difficulty [16].

The majority of previous ERP work has focused on feature selection with relatively simple stimuli composed of a small number of features. One goal of the present study was to examine the selection of different object features embedded in a single complex object, characterized by a combination of three features, none of which was spatial location. Participants engaged in a feature-based selective attention task in which they were instructed to actively discriminate the target stimulus based on a combination of color (C), orientation (O), and shape (S) features. ERP data were collected and the SN and P3 time segments were examined for the different feature combinations. With this design, we are able to compare conditions with different numbers of target versus non-target features, As an example, it is possible to calculate the ERP difference of stimuli in which all target features are present, C+O+S+, versus those in which *only* the target color is not present, but the other target features are present, C-O+S+.

Based on the previous studies as discussed above, we addressed the following experimental questions:

1. Is there distinct feature-specific sensory selection in a feature space spanned by three dimensions, or is there evidence for an all-or-none selection that occurs only for the target?
2. Is there an effect of the number of target/non-target features in the object on the amplitude, topography, and latency of the SN? Specifically, we predict a more rapid but less pronounced SN when calculated for stimuli low in the attention hierarchy (i.e. with a small number of target features).
3. Do the SN and P3 display similar sensitivity to the experimental manipulations? Specifically, the P3 is expected to reflect the “targetness” of the stimulus and not necessarily selection of isolated features.
4. What is the effect of the saliency of features on the SN and P3 components?

These questions were examined in a series of two experiments using the same basic design, but manipulating the feature saliency in a between-subject fashion.

## Materials and Methods

### Ethics Statement

All participants gave written informed consent prior to participating. All procedures were approved by the local institutional review board of the University of Florida and were in line with the Declaration of Helsinki.

### Experiment A

The first experiment examined the combined processing of color, orientation, and shape of Gabor patches in a fully crossed design. We manipulated the attention to conjunctions of those features to enable analyses of feature selection across different levels (e.g., all stimuli with target color against all stimuli with non-target color), as well as for specific combinations of target and non-target features.

#### Participants

Twenty-two right-handed undergraduate students at the University of Florida provided written consent following the guidelines proposed by the University of Florida's Behavioral/Non-Medical Institutional Review Board and received course credit for their participation. Six subjects were excluded due to insufficient completion of the task or were rejected for high artifact content. The data from sixteen participants (11 female, age range 18–21 years, mean age 19.0) with normal to corrected-to-normal color vision were included in the final analysis.

#### Stimuli and Task

**Figure 1:**
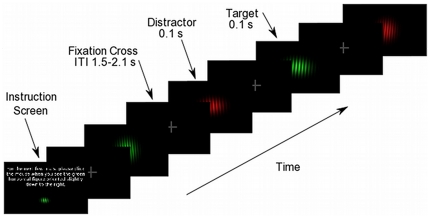
Trial sequence and stimuli. Graphical depiction of the trial sequence and a representative sample of the stimuli from Experiment A. The four enlarged Gabor patches represent the (C+O−S−), (C−O−S+), (C+O+S+) and (C−O+S−) conditions respectively, when the third presentation (C+O+S+) serves as the target stimulus. Please note the trial sequence is not drawn to scale.

Stimuli consisted of eight Gabor patches presented against a constant solid black background. Each Gabor patch was composed of 28 alternating (color/black) sinusoidal bars whose greatest contrast was at the center of the stimulus, with a Gaussian decline to the edges. Each stimulus consisted of three attributes: color, orientation, and shape. Gabor patches were either red (219, 21, 22; standard RGB values) or green (64, 240, 45). The Michelson contrast (MC), which uses the highest (L_H_) and lowest (L_L_) luminance values in a stimulus, was used to calculate the luminance contrast of the Gabor patches (MC  =  L_H_ −L_L_/L_H_ + L_L_). Luminance values were measured using a Gossen Mavo-Spot 2 luminance meter and resulted in the following luminance and contrast values for the red and green stimuli, respectively, when derived from the central bar of the Gabor patch, which had maximum brightness (L_H_ = 9.5 cd/m^2^, L_L_ = 0.05 cd/m^2^, Michelson contrast (MC) = 0.99; L_H_ = 32.9 cd/m^2^, L_L_ = 0.05 cd/m^2^, MC = 0.99). Stimulus orientation was manipulated by rotating the Gabor patch gratings relative to a vertical orientation (5° or 355°), and either a circle or an oval defined the shape of a stimulus. Each stimulus was presented for 0.1 second in the center of a 20-inch monitor situated 1.5 meters directly in front of the participants. From this distance, the stimuli subtended a visual angle of 3.5 degrees and had a spatial frequency of 4.67 cycles per degree. Target and non-target stimuli were presented sequentially in random order, with each of the eight stimuli serving as the target stimulus in one block and as a distracter in the other seven blocks. In a single experiment, each stimulus served as a target and non-target stimulus an equal number of times, relative to the other stimuli. During the interstimulus interval, ranging between 1.5–2.1 seconds, a fixation cross was present occupying 1.0° of visual angle. The experiment was organized into 8 experimental blocks, each having one stimulus as the target. Thus, a block contained one target stimulus and seven non-target stimuli, each stimulus appearing ten times throughout the block in random order. A block consisted of 80 trials, resulting in 8 total blocks and 640 total trials. At the beginning of each block, subjects were presented with an instruction screen and were instructed to attend to a target stimulus containing a specific combination of color, orientation, and shape. These features were described in writing and the actual target stimulus was presented on the instruction screen to ensure that the participants fully understood the task. The duration of each block was approximately 6 minutes. Participants were instructed to click the mouse upon detecting the target stimulus and avoid responses to non-target stimuli (go/no-go task). A graphical depiction of the trial sequence and a representative example of the stimuli (from Experiment 1) are illustrated in Figure 1. Additionally, participants were instructed to avoid head movements and to maintain gaze on the central fixation cross at all times.

### Experiment B

#### Participants

Seventeen right-handed undergraduate students at the University of Florida provided written consent following the guidelines proposed by the University of Florida's Behavioral/Non-Medical Institutional Review Board and received course credit for their participation. Two subjects were excluded from the study due to insufficient completion of the task or were rejected for high artifact content, resulting in fifteen participants (9 female, age range 18–22 years, mean age of 19.73) with normal to corrected-to-normal color vision in the final analysis.

#### Stimuli and Task

Experiment B was designed to minimize the saliency of color and enhance the saliency of orientation. The design and procedure of Experiment B were identical to those implemented in Experiment A; the duration of each block was approximately 6 minutes. Modifications were made only to the stimuli and individual stimulus descriptions. To induce a more engaging color selection process, a de-saturated red (138, 65, 23; standard RGB values; L_H_ = 5.5 cd/m^2^, L_L_ = 0.05 cd/m^2^, luminance values; Michelson contrast (MC) = 0.98) and green (102, 124, 38; L_H_ = 6.7 cd/m^2^, L_L_ = 0.05 cd/m^2^; MC = 0.99) replaced the original hues in Experiment A. Increasing the degree of rotation of Gabor patch gratings by an additional 15° from center to 20° and 340° magnified the orientation feature.

### Data Analysis

#### Electrophysiological Recordings

EEG was recorded continuously with a Geodesic Sensor Net 257-electrode array. Electrodes covered wide areas of the head, including facial and neck regions. Impedance for each electrode was kept below 60 kΩ, and the vertex electrode (Cz) was used as the recording reference. All channels were preprocessed on-line by means of a 0.1- to 90 Hz band-pass filter with a sampling rate of 250 Hz. Further processing was performed off-line.

#### Behavior

Behavioral accuracy was calculated to identify any differences in difficulty across blocks, as well as effects of fatigue. Percentage of correctly identified targets (hits), misses, and false alarms were calculated for each overall feature condition and for individual features by subject. Only reaction times over .2 and less than 1.5 seconds after target onset were considered correct responses. Reaction times longer than 1.5 seconds qualified as missed responses. This temporal range was selected on the basis of earlier studies with feature-base attention tasks [17], in which participants had median response times of around 600 ms, ranging well into the 1-second region. False alarms were calculated as the percentage of non-targets followed by a response. Differences among selection conditions were evaluated by means of omnibus repeated-measures analysis of variance (ANOVA) with factors of hemisphere (right, left), color selection (red vs. green), orientation selection (355°/340° vs. 5°/20°) and shape selection (circle vs. oval). Data were gathered between 160–300 ms for the SN and between 300–450 ms for the P3.

#### Electrocortical data: segmenting and rejection of trials

All stimuli were included in the analysis. Continuous data were digitally filtered using a Butterworth low-pass filter with a 40-Hz cutoff and 24-db attenuation at 50 Hz. Single epochs of 1100 ms in length (300 ms pre- and 800 ms post-stimulus onset) were extracted from the continuous EEG signal. These segments were submitted to a multivariate semi-automatized artifact detection procedure designed for multi-channel electrophysiology [30]. This procedure is standard in studies using the EGI dense-array system and has been validated in a plethora of published studies of EEG and ERPs [30]. A subset of electrodes located at the outer canthi and below the right eye was used to determine the horizontal and vertical electrooculogram (EOG). A combination of trial exclusion and channel approximation based on statistical parameters of the data is used to exclude channels and trials that were contaminated with artifacts. Recording artifacts were first detected using the recording reference (i.e., Cz), and then global artifacts were detected using the average reference, which was used for all analyses. Bad channels were interpolated when outlying (>2 SD above the median) the distribution with respect to amplitude, variance, and maximum differential (see Junghofer et al 2000), and a maximum of 25 channels was set for interpolation. Participants exceeding this value would be discarded, but no participant in this study failed this criterion. Subsequently, distinct sensors from particular trials were removed based on the distribution of their amplitude, standard deviation, and gradient. Data at eliminated electrodes were replaced with a statistically weighted spherical spline interpolation from the full channel set [31]. Using an interactive algorithm [30], it was ensured that extrapolated channels were not all located in a narrow region, which would render the interpolation invalid. In addition, vertical and horizontal EOG were inspected visually on the level of single trials and any remaining bad trials were rejected entirely. Participants with excessive eye movements or blinks, or more than 25 channels containing artifacts, were discarded. On average, a total of 476 trials were retained overall, with no difference between attention conditions (i.e., blocks) or experiments. Participants performing below 50% for correctly identified targets were also excluded from the final analysis (a total of 6 participants were excluded).

#### Event-related potentials

**Figure 2:**
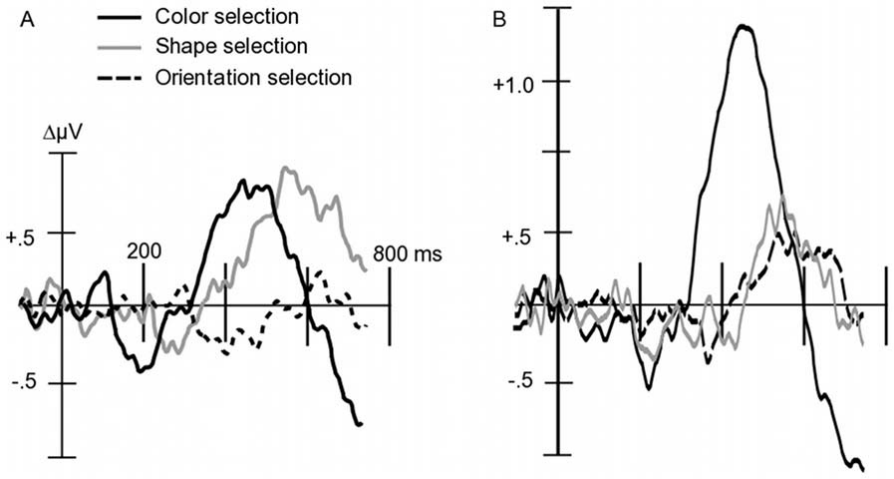
Grand mean ERP difference waveforms for target – non-target feature conditions. Grand mean (A: n = 16, B: n = 15) ERP difference waveforms for target - non-target feature conditions for (A) Experiment A and (B) Experiment B. Difference waveforms were calculated by averaging over posterior electrode sites including P9, PO7, O1, Oz, O2, PO8, P10, Iz and immediate neighboring electrodes shown with positive voltages up. The SN begins around 140–180 ms post-stimulus and continues for an additional 200 ms, resulting in a waveform shown to systematically correlate with the discrimination and selective processing of target features. The SN consistently demonstrated the greatest amplitude for color selection (target vs. non-target color), which suggested color was the most discernable feature by participants in both experiments, followed by shape, then orientation of the Gabor grating.

**Figure 3:**
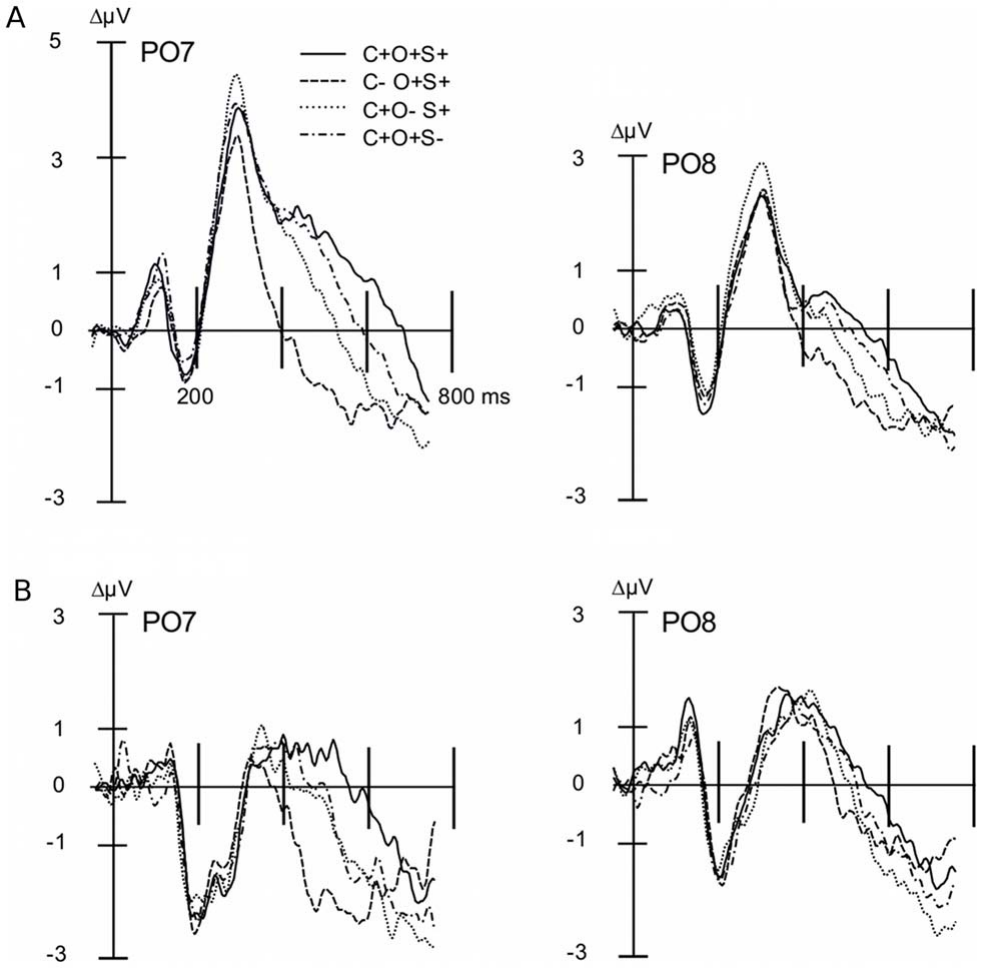
Grand mean ERP waveforms for target and non-target conditions. Grand mean (A: n = 16, B: n = 15) ERP waveforms for the target condition (all features present) and non-target conditions with attended color, orientation, and/or shape, for Experiment A (top) and Experiment B (bottom). Waveforms from two posterior electrode sites (PO7 and PO8) are shown with positive voltages up.

All potentials were evaluated using average-referenced, spline-interpolated scalp topographies. Although these topographies do not indicate the neural origin of electrocortical activity, they allow an accurate representation of the current flow on the surface of the volume conductor, i.e. the head [31]. In order to extract the SN, attentional difference waves were obtained by subtracting the ERP of a given non-target stimulus, B, from the ERP of a stimulus, A, having more target features than B [14], [15] (Figure 2). Our focus was on three main differences reflecting (i) color selection (e.g., [C+O+S+]-[C-O+S+]), (ii) orientation selection (e.g., [C+O+S+]-[C+O−S+]), and (iii) shape selection (e.g., [C+O+S+]-[C+O+S−]) (See Figure 3 for non-difference waveforms). The SN time window was selected based on previous feature-based attention work [32] and the grand mean difference waves across all subjects. A convergence of these criteria suggested a time window within 160–300 ms, further segmented into early (160–208 ms), middle (208–256 ms), and late (256–300 ms) SN time windows to examine the temporal dynamics of different conditions.

### Statistical analysis

Two statistical strategies were employed to examine the experimental questions of this study. To enhance sensitivity of the analyses of SN to systematic feature-related variations in scalp location and latency, we used permutation-corrected t-maps and F-maps to evaluate differences of spline-interpolated voltage topographies between attention conditions within the SN window. Statistical parameters were calculated at each EEG sensor and time point within the SN window for the comparisons of interest, and significance thresholds were determined for each hypothesis (below) by calculating 8,000 topographies on random permutations of the existing data, shuffled within subject but across conditions. The maximum statistic for each topography entered a reference distribution, whose 5% tails serve as the criterion for statistical significance (see [33] [34] for a similar procedure). Specifically, the following permutation-corrected tests were conducted:

(a) To address the hypothesis of feature-specific selection of individual attributes (color, orientation, shape) for the multi-feature objects used here, we compared the average maps containing all conditions in which the attended version of a given feature was present against all conditions in which that feature was unattended. Using color as an example, the unattended features (C−O+S+; C−O+S−; C−O−S+; C−O−S−) were subtracted from the attended features (C+O+S+; C+O−S+; C+O+S−; C+O−S−), resulting in the difference comparison for overall attended – unattended conditions for each feature, separately. Permutation corrected t-tests were conducted at each sensor and maps were drawn for each feature separately, highlighting the sensors showing above-threshold differences. A significant difference in this analysis favors the interpretation that the specific feature is selected for over the interpretation that three features are selected together in an all-or-none fashion.

(b) To examine whether selection of a given feature differed according to the level of attention (i.e. the number of relevant features other than the critical feature) we compared, for each feature and level separately, the individual difference maps representing the difference between conditions in which the attended version of a given feature was present against the respective condition in which that feature was unattended. For instance, in terms of color selection, there are 4 conditions in which color is attended (C+O+S+; C+O−S+; C+O+S−; C+O−S−), and the respective color unattended condition (C−O+S+; C−O+S−; C−O−S+; C−O−S−) is subtracted from each condition separately to yield difference waveforms. Permutation corrected F-tests were conducted at each sensor and maps were drawn highlighting any differences between the difference waveforms across these four levels. Significant effects in this analysis indicate that the SN varies as a function of the level of attention that is paid to the accompanying features in the stimulus.

(c) To test whether there are systematic topographical and amplitude differences between the SN deflections in response to different features, we compared the difference maps for each feature selection in a pair-wise fashion, using permutation-corrected t-maps. Significant differences indicate an amplitude and/or location difference between two difference waveforms, each reflecting selection for a particular feature. For example, in the Color – Orientation condition, target – non-target comparisons were calculated separately for both color and orientation (see description in [c]), and the resulting difference waveforms were used in the Color – Orientation pair-wise feature selection condition.

(d) We employed the same evaluation of systematic topographical and amplitude differences of the SN deflections using difference map comparisons and permutation-corrected t-maps to address potential feature effects between Experiments A and B. Significant differences are indicated in the permutation maps by means of amplitude modulation in the context of Experiment A – Experiment B for color, orientation and shape, respectively. (To be discussed in joint analysis of Experiments A and B.)

It is important to note that these topographical tests do not indicate the location of neural activity. Furthermore, because of the high number of electrodes and the use of the average reference, it is possible that voltage differences appear at locations outside classical regions, typically located over brain tissue. This is the consequence of the well-known effects of (a) projection of deep sources to remote electrodes and (b) smearing of the scalp voltage distribution by volume conduction, among others. We opted for showing the voltage projection on a realistic head model to enable readers to assess potential sources and the quality of the signal overall, with respect to an anatomically meaningful reference, i.e., a head model.

**Figure 4:**
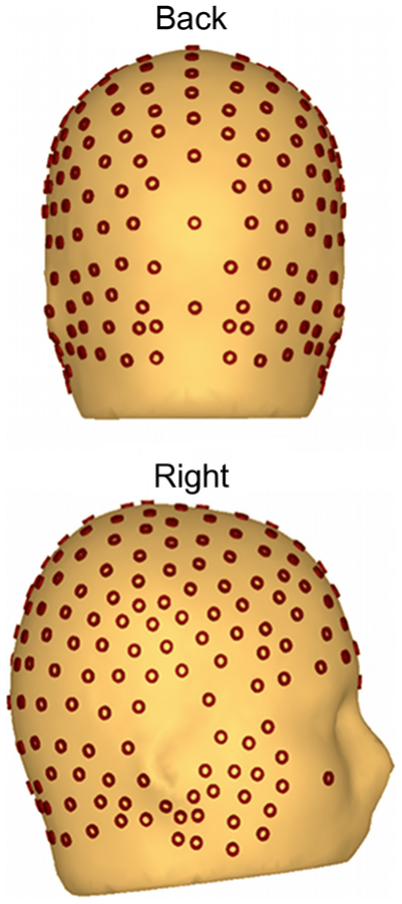
Geodesic Sensor Net 257-electrode array. Layout of the Geodesic Sensor Net 257-electrode array used for analysis in the current study. Back and right side views are shown.

For statistical analyses of the P3, a subset of electrodes was used spanning the parietal region where this signal was maximal (including CPz, Pz, O1, POz, O2, and their nearest neighbors). Voltages were averaged across this electrode cluster and across the time range between 300–450 ms, to result in a measure of P3 amplitude. The locations of these electrodes are indicated schematically in Figure 4. Analysis of the overall effects of experimental manipulations across all conditions were evaluated by means of omnibus repeated-measures ANOVAs with factors of color selection (red vs. green), orientation selection (355° vs. 5°) and shape selection (circle vs. oval).

## Results

### Experiment A

#### Behavioral Data

The percentage of correct target detections ranged from 52.5% to 96.3% in different subjects, with an overall average of 80.2% for correctly identified targets. False alarms were calculated as the percentage of non-targets followed by a response (12.4%). Hits, misses, and false alarms were compared across the 8 feature combinations (each of which served as the target in one experimental block). As expected, the overall hit rate (M = 80%, SEM  = 1.4%) did not vary as a function of feature combination (F(1, 15)  = 0.085, n.s.), nor did any other behavioral measure (Misses: F(1,15)  = 0.067, n.s.; False Alarms: F(1,15)  = 0.015, n.s.; Correct Rejections: F(1,15)  = 0.013, n.s.).

#### Electrophysiological data

**Figure 5:**
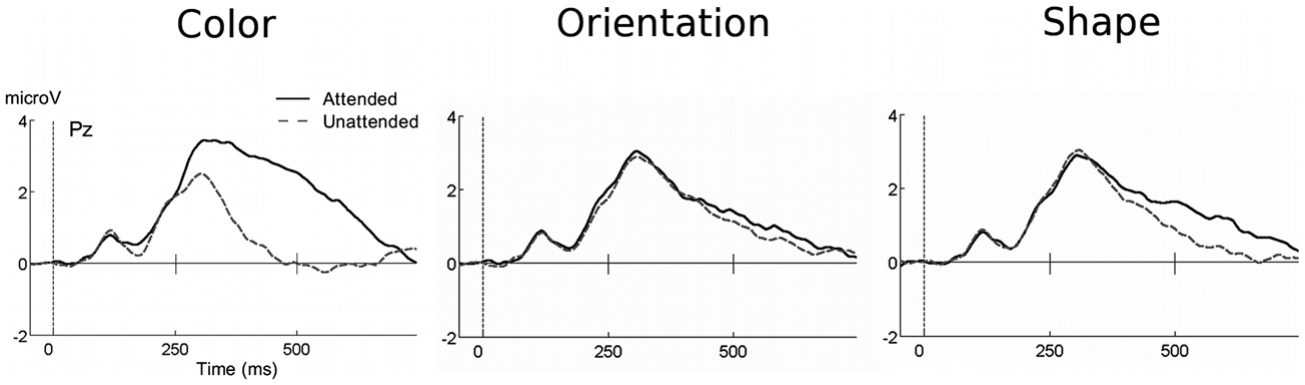
Grand mean ERP waveforms of target and non-target feature conditions. Grand mean, n = 16, ERP waveforms for target and non-target feature conditions in Experiment A.

Reliable visual ERP waveforms were extracted from all individuals and displayed the expected sequence of deflections (see Figure 5). In a first step, we examined the P3 amplitude in the time range of 300–450 ms. P3 amplitude was modulated by the experimental manipulations of target versus non-target stimulus features. The P3 displayed maximal amplitude for stimulus conditions only in which the target color was present (Figure 5, left panel), resulting in a color main effect (F(1, 15)  = 11.763), p<0.005).

**Figure 6:**
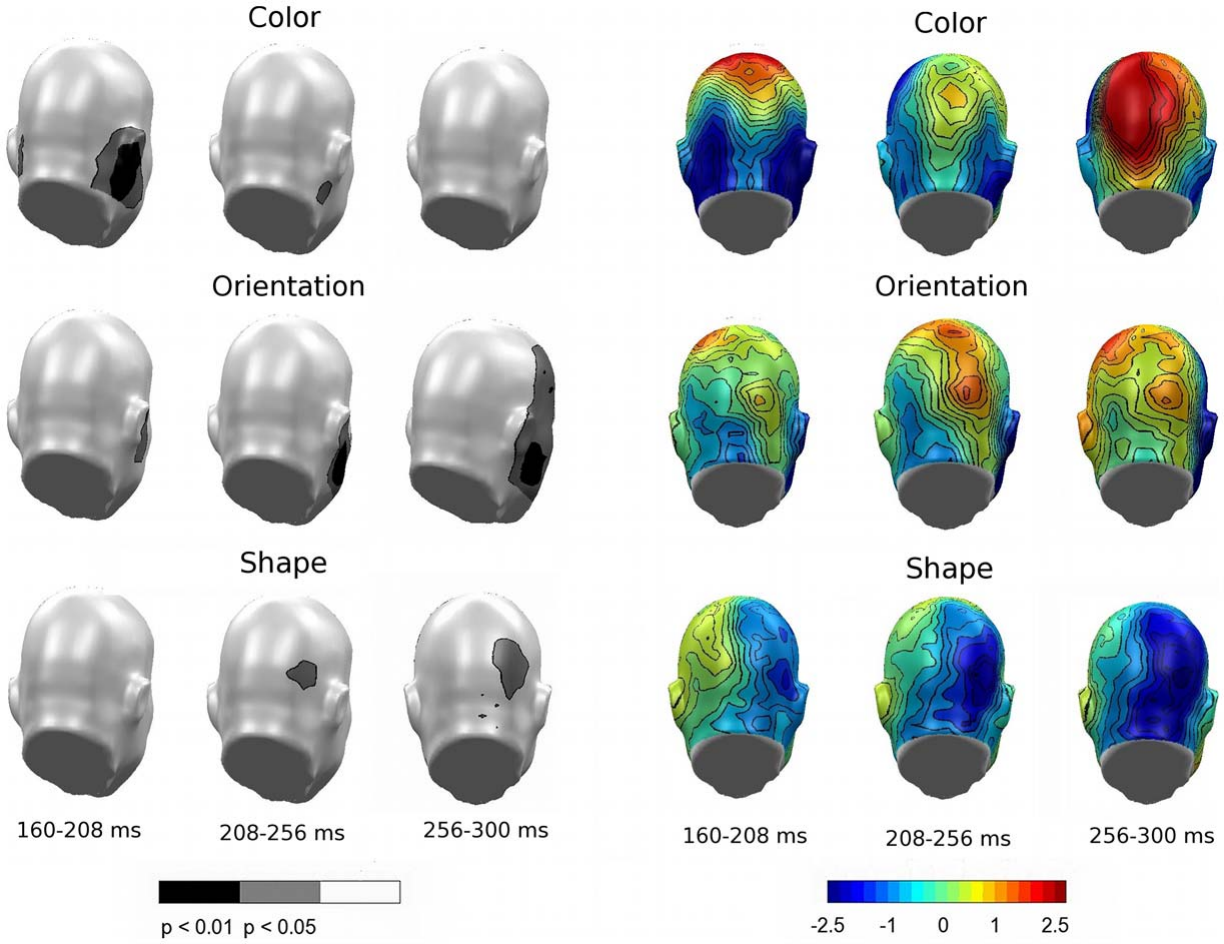
Feature selection difference topographies, Experiment A. Permutation-corrected t-maps (left panel) and difference topographies (right panel) containing all conditions in which the target feature was presented against the respective non-target feature in Experiment A. Significant regions highlight feature-specific selection for a give attribute.

Permutation tests in the SN time window first focused on the question as to whether selective processing took place for each feature separately. As shown in the permutation-corrected t-maps (left panels of Figure 6) for three subsequent time windows in the SN range, selection effects were found at posterior sensors as expected (p_perm_<.01 shown in black). Generally, differences were more pronounced at right-hemisphere sensors for all features, but differed in the timing of the selection process. Color selection occurred rapidly in the earliest SN time range, highlighted by the typical bilateral SN pattern seen most clearly in the top left topography (160–208 ms) in Figure 6. Selective processing for orientation and shape were relatively delayed, extending through the entire SN time range. All features were related to specific SN modulation in the critical time window of 160–300 ms, supporting notions of feature-specific sensory selection.

A second step examined effects of the number of target features to determine the possibility of a selection advantage for the presence/absence of specific feature combinations. No significant differences were observed, implying there is no variance in the SN as a function of the number of target features.

**Figure 7:**
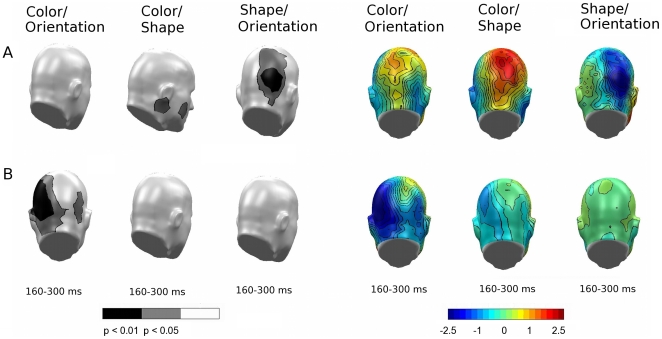
Comparison of feature-selection topographies, Experiments A and B. Permutation-corrected t-maps (left panel) and topographical maps (right panel) used to compare the differences of SN deflections in response to pair-wise feature selections for Experiment A (top) and Experiment B (bottom). Significant regions highlight systematic difference in the SN deflections in response to different features.

In a third step attempting to address differences in SN deflection pertaining to within-experiment feature selections (Figure 7), selection to color showed a slight advantage over shape, exhibiting more overall positivity (topography; top panel, center) and respective significant differences (permutation-corrected t-map; top panel, center) in the lower right hemisphere. Additionally, orientation was associated with more negativity than shape (topography; top panel, right), resulting in a broad area of significant differences at right hemispheric recording sites (permutation-corrected t-map; top panel, right). Such differences in topography and amplitude yield support for systematic differences in the SN deflections in response to different features.

The use of highly salient colors in Experiment A suggested strong color selection effects on the SN and minimal discrimination of orientation and shape. This led to a second study, which followed identical procedures but incorporated a revised stimulus set with variations to color and orientation. In an effort to reduce potential color pop-out effects and enhance the saliency of feature orientation, the second experiment was designed to involve stimuli with de-saturated color and increased angular orientation.

### Experiment B

#### Behavioral Data

Data reduction followed the same procedures used in Experiment A. The percentage of correct target detections averaged 87.0%, ranging from 75.0% to 98.8% in different subjects. The percentage of false alarms was 7.47%. Consistent with results from Experiment A, the overall hit rate (M = 86.9%, SEM  = 0.69%) did not vary as a function of feature combination (F(1, 14)  = 2.148, n.s.), nor did any other behavioral measure (Misses: F(1,14)  = 2.148, n.s.; False Alarms: F(1,14)  = 1.275, n.s.; Correct Rejections: F(1,14)  = 1.275, n.s.).

#### Electrophysiological data

**Figure 8:**
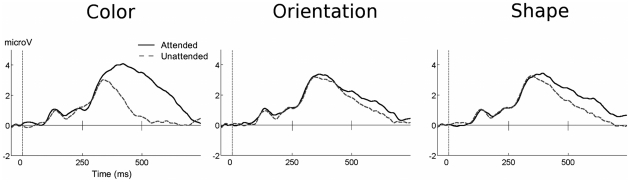
Grand mean ERP waveforms of target and non-target feature conditions. Grand mean, n = 15, ERP waveforms for target and non-target feature conditions in Experiment B.

Amplitude of the P3 was examined as in Experiment A. Figure 8 (left panel) shows that the maximal amplitude between 300–450 ms was seen for the stimulus conditions in which the target color was present. Paralleling Experiment A, the P3 demonstrated a color main effect between 300–450 ms (F(1, 14)  = 9.522), p<0.05). In addition, a significant orientation main effect (F(1, 14)  = 6.625, p<0.05) and Orientation x Shape interaction (F(1, 14)  = 7.893, p<0.05) were present in the same time range. This suggests that in Experiment B, the P3 tends to more strongly index the targetness of a particular feature combination, compared to Experiment A.

**Figure 9:**
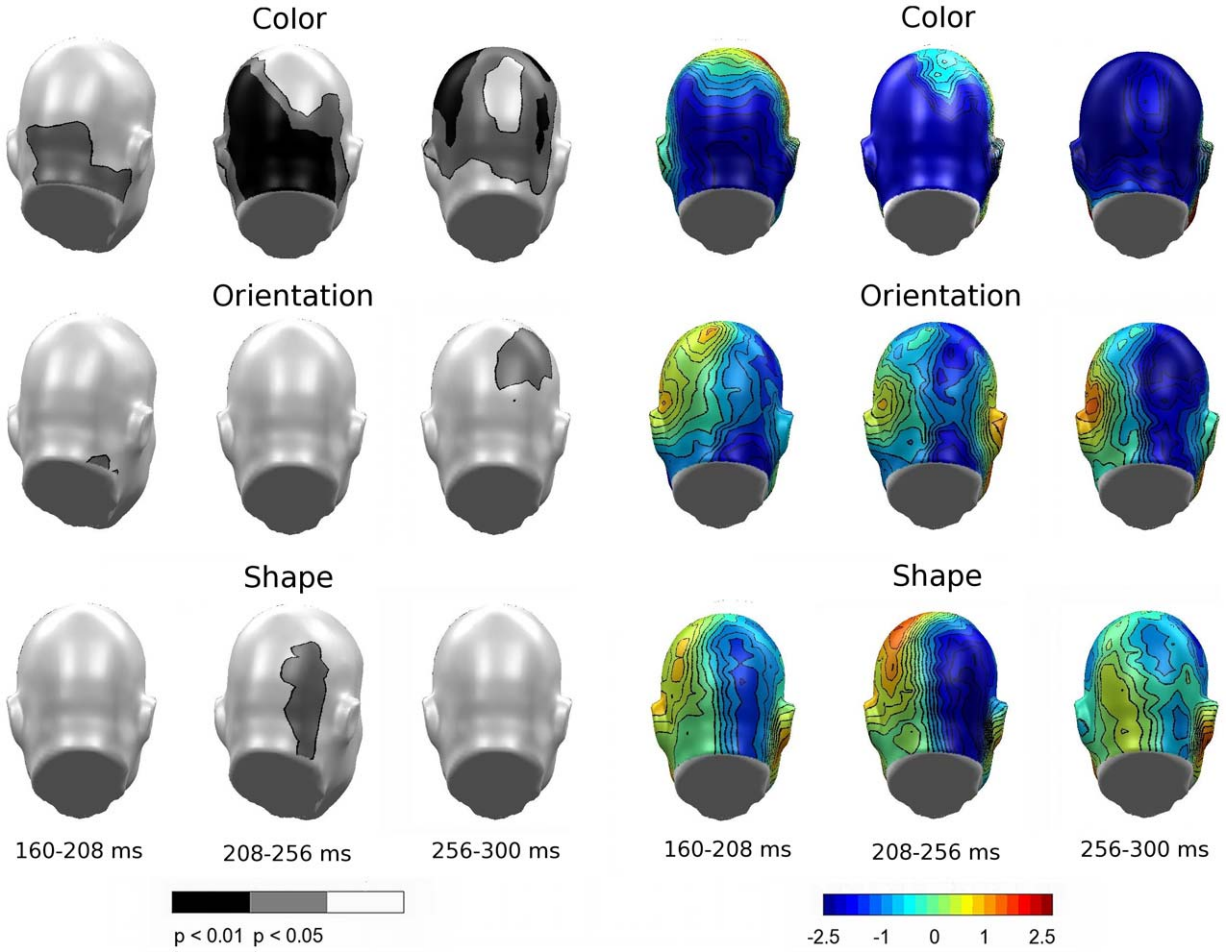
Feature selection difference topographies, Experiment B. Permutation-corrected t-maps (left panel) and difference topographies (right panel) containing all conditions in which the target feature was presented against the respective non-target feature in Experiment B. Significant regions highlight feature-specific selection for a given attribute.

Permutation tests were also conducted for Experiment B to address the same hypotheses tested in Experiment A. Consistent with results from Experiment A, selection effects (p_perm_<.01 shown as black scalp regions in black in Figure 9) were found at posterior sensors, predominantly in the right hemisphere. As evidenced in Figure 9, less salient coloring resulted in an extended SN period demonstrating significant negativity in all selection windows, with the most pronounced difference for color selection occurring between 208–256 ms (top, center topography). Salient orientation resulted in an overall pronounced negative difference topography as well (Figure 9; center topography panel), and this difference reached the permutation corrected significance threshold at right hemisphere-sensors between 256–300 ms (permutation-corrected t-map; second row, right). As expected and paralleling Experiment A, shape selection remained consistent throughout the SN time range.

In a second analysis evaluating selection effects for different numbers of target features, the data did not support such an advantage for the presence/absence of specific feature combinations (all p_perm_>.1, figure not shown). When examining whether the SN topographies were different for different feature types, the posterior SN amplitude was more negative for color than for orientation, which resulted in an area of statistical significance in a bilateral pattern most heavily concentrated in the left hemisphere (see Figure 7, bottom left), but no difference was observed for the other feature comparisons.

By incorporating the new stimulus set with reduced color saliency and enhanced differences in directionality, participant performance increased by approximately 7% in correctly identified targets. Even with the reduced saliency of color and the associated shifts in latency, amplitude of the P3 was still strongly determined by the presence or absence of the target color, suggesting the important role of color in target selection and response preparation. Findings with the SN suggested early color salience, even with de-saturated color, and greater salience of color than orientation, despite heightened saliency of orientation. In an attempt to examine the reliability and specificity of findings across the two studies, we performed a joint analyses, which capitalized on the fact that shape was held constant across the two experiments, thus representing a control condition, sensitive to noise or spurious differences between samples.

### Joint analyses: Experiments A and B

#### Behavioral Data

As expected from the previous behavioral data, significant differences in hit rate (Study A: M = 80%, SEM  = 1.3%; Study B: M = 86.91, SEM  = 0.7%) for Experiments A and B were observed across all feature conjunctions (F(1, 14)  = 8.413, p<0.005).

#### Electrophysiological data

In a between-studies examination of the P3 amplitude between 300–450 ms, clear latency differences in peak amplitude exist. Referring to Figures 5 and 8, respectively, Experiment A demonstrated earlier P3 effects, whereas the P3 for Experiment B was most prominent in the later segments. Such evidence for an early P3 in Experiment A can also be seen in Figure 6.

**Figure 10:**
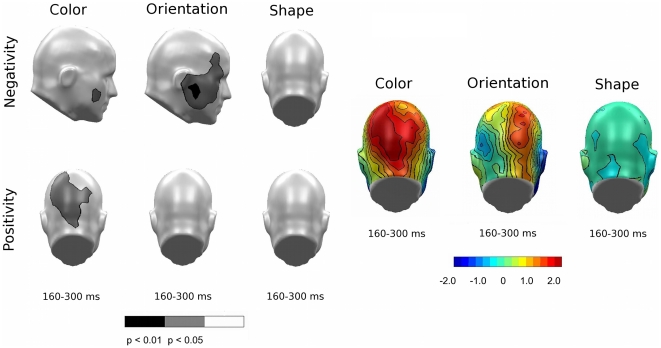
Comparison of feature-selection topographies between experiments. Both panels show the overall feature comparisons for the individual feature effects between Experiments A and B. Left panel: Permutation-corrected t-map. Topographical distribution of the statistical significance of feature selections between Experiments A and B in the form of negative (top) and positive (bottom) voltage differences. Right panel: Difference topography of overall feature comparisons (A–B).

Analysis of feature-type related effects on SN topography between experiments yielded a significant difference in the feature attributes of color and orientation, as suggested by the behavioral data. Figure 10 illustrates the difference between Experiments A and B (A–B), showing sensors with significantly different topographies in the SN time range for negative and positive voltage differences. The relatively quick selection process for color resulted in greater early positivity during color selection for Experiment A (Figure 10, left topography), and also reflected a region of significance (Figure 10, permutation-corrected t-map; bottom left) in the occipital and posterior parietal cortices. This difference is also consistent with a more negative and prolonged selection process for color in Experiment B (see also Figure 9 topography; top panel). Likewise, enhanced orientation resulted in more negativity produced by Experiment B (Figure 10, center topography), thus resulting in a statistical difference between the two stimulus sets regarding orientation (Figure 10, permutation-corrected t-map; top center). Note that no difference between experiments was observed for the shape feature, which was unchanged across the two experiments.

## Discussion

The overall goal of the present series of experiments was to study feature selection in multidimensional stimuli, with targets defined by a combination of three distinct characteristics. Two electrophysiological measures were considered: the SN, a measure of early attentional selection, and the P3, a measure of higher-order processing of target versus non-target information. Consistent with previous work [1], [35], the SN showed the expected pattern of amplitude enhancement for each of the target features (color, orientation, shape) when considered across the respective other features. Contradicting expectations, both experiments failed to observe any evidence of integrative processing on the level of the SN. Combined processing would be reflected in effects of attending/ignoring the concurrent features (e.g. shape) on the SN to a given feature (e.g. color). Such interaction effects were observed in earlier work in lower dimensional feature spaces [17], but were absent in both experiments described in this report.

Comparing the electrophysiological response across features, the SN consistently demonstrated the greatest amplitude for color selection (target vs. non-target color), which suggested that color was the most discernable feature by participants in both experiments, followed by shape, then orientation of the Gabor grating. Such an order is consistent with findings from psychophysics [36] and electrophysiological studies on object recognition [37]. The temporal sequence of significant differences between target and non-target conditions (i.e., of the SN) suggested that relatively more salient features are selected earlier and are accompanied with more pronounced SN waveforms. For instance, reducing the saliency of color while enhancing orientation differences led to heightened latencies for the color SN as well as greater orientation SN. The selection of shape, which was unaltered between the two experiments and thus served as a control in the between-experiment comparisons, did not display any cross-experiment changes. These findings are in line with predictions of models of feature-based attention such as the feature-similarity-gain model [38], which surmises that attention to a specific feature enhances electrocortical processes specific to that particular feature in different areas of visual cortex, inside and outside of specific spatial receptive fields. Models that assume that attention acts upon the sensory process involved in the extraction of a given feature would be consistent with the present finding that color selection appears to be faster and more pronounced, when color is more salient. Such improved effects of attention with changes along a saliency dimension have been reported for macaque cortex as well [39].

In addition to differences between features within each experiment, manipulating feature saliency between experiments resulted in localization and latency differences of the SN components. Experiment A demonstrated early selection for color, co-occurring with the ability of participants to easily discriminate between target and non-target stimuli. Of the two more difficult feature distinctions in Experiment A, shape discrimination was associated with a more consistently negative ERP difference, extending through the entire SN time range. Enhancing the difference in the orientation of the Gabor grating (Experiment B) resulted in a more pronounced electrophysiological selection process, associated with the greatest negativity in the latest SN time window chosen for analysis. Shape, which remained unchanged between experiments, was the most consistent feature in both negativity and latency, experiencing no significant variation between studies. The consistency of shape throughout Experiments A and B adds validity to the notion that differences between the two experiments are due to adjustments in stimulus saliency and not simply to random differences in participant characteristics.

Across experiments, differences were consistently more pronounced on the right hemisphere for all features in both experiments. In addition, SN differences were consistently seen at posterior sensors, with inferior sites showing greater sensitivity to color selection, and more parietal sites generally showing sensitivity to shape and orientation selection. These differences are consistent with reports that highlight the role of specialized sensory cortices in the selective attention allocated to one specific feature [9]. However, occipitocortical activation is not necessarily projected only to posterior sensors, but may also be projected to other regions of the scalp (as seen in Figure 10, permutation-corrected t-map; top panel, center). It is important to note that the scalp-recorded EEG represents the underlying voltage gradients, which may be altered as they pass through the volume conductor (i.e. body). These electrocortical gradients are sensitive to tissue properties between the electrical source and the recording electrode on the scalp, an individual electrode's conductive properties, as well as the orientation of the cortical generator to the recording electrode. Because the present study did not attempt to localize the cerebral sources underlying ERP modulations observed at the scalp level, conclusions regarding potential generator differences for structure versus color selection effects are not warranted. It should be noted however that research with hemodynamic imaging data also supports area-specific modulation of the response in cortical regions that are sensitive to a particular feature such as color or motion [40].

The SN component reliably reflected feature selection of a single target, across all other conditions, across different levels of attention paid to other features, and across different feature conjunctions, which suggests that the presence of non-target features did not affect the SN amplitude. As noted above, the SN amplitude showed spatial specificity, also suggesting that selection effects in a specific area of cortex might not interact in the time window indexed by the SN. This would be in line with earlier notions that have argued that effects of competition and integration of features across different feature dimensions happens at a later stage and is best examined using measures of oscillatory activity [32]. Such findings have observed dissociations with SN and subsequent gamma power modulations in the human EEG, suggesting that high-frequency oscillatory activity is enhanced for stimuli sharing the overall gestalt with the target [17], thus making it a better indicator of integrative processing.

The P3 amplitude in the present study showed differential sensitivity to feature saliency and provided information complementary to the SN. Consistent with previous research [29], the P3 was enhanced only for stimuli containing the target color in experiment A, regardless of the presence (or absence) of other task-relevant feature attributes. Experiment B, with a more balanced saliency of feature attributes, indicated that the P3 was sensitive to feature conjunctions, being greatest for the specific target stimulus, and not generally enhanced for the most salient feature.

Together with the aforementioned results, this data, in conjunction with other human [41] and animal data [42], [43], verifies that early color processing results in SN most prominent in the occipital region. Previous data [44], [45] also support the hierarchy of feature processing seen in the current study, demonstrating the ability of human subjects to rapidly and accurately identify changes in color apart from variations in other stimulus attributes of multi-feature stimuli. What remain unclear are the implications of such information on human interactions in naturalistic settings. Such information regarding processing of color, orientation, and shape can be applied to research designs incorporating real world scenarios in an effort to gain greater understanding of the mechanisms behind compound, multi-feature stimuli in realistic settings.
